# The emerging role of Cardiovascular Magnetic Resonance in the evaluation of hypertensive heart disease

**DOI:** 10.1186/s12872-017-0556-8

**Published:** 2017-05-23

**Authors:** Sophie Mavrogeni, Vasiliki Katsi, Vasiliki Vartela, Michel Noutsias, George Markousis-Mavrogenis, Genovefa Kolovou, Athanasios Manolis

**Affiliations:** 1 Onassis Cardiac Surgery Center, 50 Esperou Street, 175 61 P.Faliro, Athens, Greece; 2 Hippokration Hospital, Athens, Greece; 3 Department of Cardiology, Pneumonology and Intensive Care Medicine, Clinic for Internal Medicine I, University Hospital Jena, Friedrich-Schiller-University, Jena, Germany; 4 Asklipiion Voulas, Athens, Greece

**Keywords:** Hypertension, Cardiovascular magnetic resonance, ECG, Echocardiography, Coronary angiography

## Abstract

**Background:**

Arterial hypertension is the commonest cause of cardiovascular death. It may lead to hypertensive heart disease (HHD), including heart failure (HF), ischemic heart disease (IHD) and left ventricular hypertrophy (LVH).

**Main body:**

According to the 2007 ESH/ESC guidelines, the recommended imaging technique is echocardiography (echo), when a more sensitive detection of LVH than that provided by ECG, is needed. Cardiovascular Magnetic Resonance (CMR), a non-invasive, non-radiating technique, offers the following advantages, beyond echo:

a) more reliable and reproducible measurements of cardiac parameters such as volumes, ejection fraction and cardiac mass b) more accurate differentiation of LVH etiology by providing information about tissue characterisation c) more accurate evaluation of myocardial ischemia, specifically if small vessels disease is present d) technique of choice for diagnosis of renovascular, aortic tree/branches lesions and quantification of aortic valve regurgitation e) technique of choice for treatment evaluation in clinical trials.

The superiority of CMR against echocardiography in terms of reproducibility, operator independency, unrestricted field of view and capability of tissue characterization makes the technique ideal for evaluation of heart, quantification of aortic valve regurgitation, aorta and aortic branches.

**Conclusions:**

CMR has a great potential in early diagnosis, risk stratification and treatment follow up of HHD. However, an international consensus about CMR in HHD, taking under consideration the cost-benefit ratio, expertise and availability, is still warranted.

## Background

### Alterations occurring in hypertensive heart disease (HHD) and the role of non-invasive cardiac imaging

Arterial hypertension is the commonest cause of cardiovascular death. It may lead to hypertensive heart disease (HHD) including heart failure (HF), ischemic heart disease (IHD) and left ventricular hypertrophy (LVH). There is no consensus among new HTN practice guidelines as to target treatment of blood pressure (BP) among various subpopulations of patients. However, most guidelines now target a BP < 150/90 for patients >80y, a BP < 140/90 for patients with diabetes or CVD and a BP < 130/80 if diabetes, albuminuria, or high stroke risk is present. In United States, 1 out of every 3 adults has high BP. About 69% of people with first heart attack, 77% with first stroke and 74% with HF have BP higher than 140/90 mmHg [1].

Essential hypertension accounts for 90% of adult cases and secondary causes of hypertension for the remaining 10%. According to the Framingham Study, hypertension accounts for about 1/4 of HF cases, and the risk of HF is increased by 2-fold in men, and 3-fold in women, respectively. Finally, hypertension affects other target organs including kidneys, eyes and peripheral arteries [2].

Left ventricular hypertrophy (LVH) is the response of myocytes to various stimuli leading to myocytes’ hypertrophy, which occurs as a compensatory response to increased afterload [2]. It is defined as an increase in LV mass, assessed by postmortem measurements, electrocardiographic (ECG), echocardiographic and Cardiovascular Magnetic Resonance (CMR) criteria. Early echocardiographic studies defined LVH as an absolute LV mass (LVM) exceeding 250 g [2]. Regression of LVH with antihypertensive treatment reduces the risk of stroke, myocardial infarction and all-cause mortality [2]. There are two main patterns of LVH: a) concentric and b) eccentric LVH [2]. Concentric LVH is considered, when LV mass increases by wall thickening in response to pressure overload, as often in middle aged and elderly patients, is associated with lower cardiac output and predicts poor prognosis. There is a pathway from hypertension to concentric LVH without focal scar [3], hypertension to concentric LVH with focal scar [4], concentric remodelling with myocardial infarction assessed by replacement fibrosis [5], and concentric LVH with symptomatic vascular events and heart failure either with replacement scar [6] or without [7, 8]. Diastolic dysfunction and/or heart failure with preserved ejection fraction (HFpEF), due to remodelling of the extracellular matrix and increase in LV filling pressures, are common in concentric LVH [9–14]. In eccentric LVH, there is an increase in LV mass without increased concentricity and is associated with higher cardiac output [8] (Fig. 2). It has not been fully clarified why patients develop a specific LVH pattern, as a response to hypertension. Factors such as pressure, volume overload, ethnicity, gender, obesity and plasma renin levels, all seems to play a role [2]; however, the clinical implications of various LVH patterns are still under evaluation. The aim of this review is to discuss the potential advantages and disadvantages of CMR over the currently used echocardiographic techniques and clarify its additive value in the evaluation of HHD.

### “Needs and wants” in HHD evaluation

Various non-invasive techniques have been used to elucidate the pattern of HHD, including HFpEF. ECG and echocardiography were for many years the only techniques for evaluation of HHD. Although ECG measures of LVH were associated with cardiovascular disease risk in the Framingham study [15], the ECG evaluation of LVH lacks sensitivity and specificity, particularly in young male patients [16, 17].

Recently, a discrepancy documented in diagnostic performance and agreement on predictive ability suggests that LVH by ECG and LVH by CMR are likely to be two distinct phenotypes [18].

Echocardiography has been successfully used in clinical trials and provided important knowledge in HHD [19]. LVM assessment is useful in severe LVH; however, due to high variability, it underscores patients with mild concentric, eccentric LVH and/or concentric remodelling. Additionally, a large patients’ sample is required to document LVM regression, using M-mode or 2D echocardiography, due to high inter-observer and inter-study variability [20].

CMR, due to its excellent reproducibility, unrestricted field of view and non-invasive, non-radiating tissue characterization, became a powerful player for early diagnosis and treatment assessment of HHD and gender-specific values according to age and body surface area have been already published [21]. The comparison between new echocardiographic techniques and CMR showed that the assessment of LV volumes/LVEF by echocardiography and CMR have good correlations. However, the inter-technique agreement of absolute LV volumes revealed considerable differences, with significant underestimation of volumes and LVEF with respect to CMR [22]. Another study evaluating if LVM by real-time, 3-dimensional echocardiography (RT-3DE) corresponded to CMR in patients with LVH, showed that LVM by RT-3DE correlated with that determined by CMR better than that determined by 2DE, which means that RT-3DE can overcome some of the disadvantages of 2DE in the evaluation of LVM [23]. However, another study, evaluating the accuracy of LVM calculation using new echocardiographic techniques in comparison with CMR in ischemic (IC) and nonischemic cardiomyopathy (non-IC), documented that although more accurate and reliable echocardiographic measurement of LVM was achieved by 3DE, underestimation and variability remained challenges in IC [24]. Finally, a recent study, evaluating 40 patients by echocardiography using 4 imaging modalities (M-mode fundamental imaging [FI], M-mode harmonic imaging [HI], two-dimensional [2D] FI and 2D HI) and CMR, showed that HI overestimates LVM, compared with FI and CMR leading to overestimation of prevalence of LVH in hypertensive patients. HI improves inter-observer reproducibility of LVM measurements, compared with FI, leading to a significant decrease in the number of patients required for clinical trials of LVM regression. Finally, the accuracy of LVM measurements by echocardiography is affected by LV geometry [25, 26].

Speckle tracking (ST) alone or combined with tissue Doppler imaging (TDI) seems to be suitable for measurements of regional myocardial deformation and shows better agreement with CMR tagging for regional myocardial strain than measurements based solely on TDI; however, the clinical significance of the detected differences between the methods needs to be further established [27].

### CMR “pearls and pitfalls” in the evaluation of HHD

According to the 2007 ESH/ESC guidelines the recommended imaging technique is echocardiography, when a more sensitive detection of LVH than that provided by ECG is needed, particularly in patients in whom organ damage is not detected by ECG, and in the elderly, in whom cardiac hypertrophy is frequent [28]. Additionally, in the 2010 ACCF/ACR/AHA/NASCI/SCMR Expert Consensus Document no recommendation about the usefulness of other techniques, including CMR in the assessment HHD, was discussed [29].

CMR provides a comprehensive non-invasive, non-radiating evaluation of HHD, including accurate and reproducible assessment of biventricular function, valvular disease, inflammation and stress myocardial perfusion-fibrosis. Its extensive application has the potential to lead to better understanding of pathophysiology of HHD, promoting more accurate risk stratification and personalised treatment. However, the extensive application of CMR is hampered by serious limitations, such as long examination time, lack of availability and expertise, time consuming post-processing and high cost. As any diagnostic technique, CMR carries various limitations. Scanning patients with metallic clips, pacemakers and other non CMR conditional cardiac devices is not indicated, due to safety reasons. Furthermore, paramagnetic contrast agents can not be used in patients with reduced glomerular filtration rate (GFR) < 30 mL/min/1.73 m2, due to risk of nephrogenic systemic fibrosis (NSF) [30], a scleroderma-like disease affecting the skin and internal organs [31]. If GFR is normal, its incidence is less than 1% [32]; however, in cases with reduced GFR, gadolinium should be given, only if: a) the expected benefits counterbalance the risks, b) using the lowest possible dose and c) avoiding the repeated use.

### CMR sequences needed for evaluation of HHD

Specific sequences for cardiac evaluation include steady state free precession (SSFP) cines for function and wall motion assessment, phase contrast sequences for velocity evaluation, T2-weighted short-tau inversion recovery (STIR) for oedema assessment, T1- and T2-weighted fast spin-echo for tissue characterisation, T1- weighted perfusion and myocardial late gadolinium enhancement (LGE) sequences and 3D–magnetic resonance angiography (MRA). Other more sophisticated CMR techniques such as myocardial tagging, T1, T2 mapping are at the moment part of research in HHD. Currently, 3D–gadolinium enhanced MR angiography (MRA) is considered the imaging technique of choice for evaluation of renovascular hypertension and MRI is also excellent to localize tumours leading to pheochromocytoma and primary aldosteronism. However, at the moment, no specific indications for CMR in HHD have been proposed [33].

### Comparison with other imaging techniques

The main imaging technique for comparison with CMR remains echocardiography, since both computed tomography (CT) and nuclear techniques are not included in the routine evaluation of HHD and are used only if there are specific indications. Compared with these techniques CMR has the following advantages:

#### High reproducibility of measurements

CMR is more reproducible than both M mode and 2D echocardiography for the estimation of LVM, because it does not require geometric assumptions, according to data validated by animal studies [24–36]. It provides an excellent contrast between blood and myocardium and has high spatial resolution, leading to accurate definition of endocardial and epicardial contours. Steady-state free precession (SSFP) cine imaging is the sequence of choice for measuring LVM by CMR. Usually, the absolute values of LVM by CMR are lower compared with echocardiography, because a) SSFP allows the visualization and inclusion of myocardial trabeculations in the LV volume with simultaneous exclusion from mass calculation b) the echocardiographic evaluation is based on geometric assumptions that is not the case for CMR

#### Easier and faster evaluation of treatment

CMR or 3D echocardiography are the techniques of choice in trials evaluating LVM regression, because they allow the accurate detection of small changes of LVM in small patients’ cohorts, particularly when recruitment of large patients’ numbers is not feasible. CMR, due to its high reproducibility, can demonstrate reduction in LVM and volumes with normalization of LVEF after a short period of better blood pressure (BP) control using appropriate medication [37, 38] and has been used to assess LVM regression after treatment of HHD in various studies including:

- the LIFE substudy in which the effect of high BP to ventricular remodelling was assessed [39]
- the TELMAR study [40], which compared the effect of telmisartan to metoprolol on LVH in uncontrolled hypertension
- the LVH-4E [41], in which eplerenone was compared to enalapril or a combination of both in LVH regression in hypertensive patients
- the ALIVE study [39], in which benazepril with either amlodipine or a diuretic was evaluated and recently
- the ALLAY trial [42], in which aliskiren, a direct renin inhibitor, was proved to be as effective as losartan for reduction of LVM.

#### Evaluation of intramyocardial function

CMR tissue tagging allowed the non-invasive assessment of intramyocardial displacement / strain by monitoring motion of specific material points spread in the myocardium [43–47]. The application of this technique in large epidemiologic studies such as the Multi-Ethnic Study of Atherosclerosis (MESA) [48] has enabled to investigate the nature of atherosclerosis in a total of 1184 asymptomatic participants (aged 45–84 years). Regional LV function was quantified by evaluating peak systolic circumferential strain (Ecc). The study proved that higher diastolic blood pressure (DBP) was associated with decreased regional LV function in asymptomatic individuals [49] and was significantly attenuated after controlling for LVM. Furthermore, LV torsional deformation was greater in hypertensive patients, despite that they had lower circumferential shortening, because torsion in hypertension with concentric remodeling is a compensatory mechanism to maintain LVEF [49].

In contrast, the echocardiographic evaluation of LV deformation is a geometry-based index, derived from linear measurements of the posterior and the septal wall and cannot distinguish between septal and posterior wall function. Thus, it is unknown, whether depressed LV deformation represents global or regional intrinsic depression of LV myocardial function in hypertrophy, due to pressure-overload [50]. In addition, the assessment of torsional deformation by echocardiography is methodologically challenging, because the distance between the basal and the apical short-axis slices can not be accurately assessed by this technique [50].

#### Diastolic dysfunction

Diastolic dysfunction is the earliest expression of HHD [51, 52], affects approximately 50% of hypertensive patients worldwide [52], correlates with the degree of LVH [53, 54] and is remarkably improved in cases with LVH regression (54). Currently, the most widely applied technique to assess LV diastolic function is the evaluation of transmitral inflow or pulmonary venous flows using Doppler echocardiography [55–58]. However, echocardiography measures the impact of altered LV diastolic properties by evaluating diastolic flow velocities, due to pressure gradient changes at the mitral orifice and flow velocities in the pulmonary veins and is unable to evaluate LV relaxation directly. Furthermore, the conventional Doppler measurements are very much load-dependent and can change dramatically during minimal alterations in heart rate and/or ventricular preload [58]. This defect can be overcome by the application of Tissue Doppler Imaging (TDI), which measures early diastolic mitral annular velocity (Ea) and late, due to atrial contraction, diastolic mitral annular velocity (Am) [59]. Ea of the lateral basal part of LV does not change significantly and any consequence, due to preload change, can be corrected by a ratio of E/Ea [59]. Additionally, 2D–speckle imaging provides a direct angle- and geometry-independent measure of circumferential strain (ε) [60, 61].

There is an excellent agreement between CMR and transthoracic echocardio-graphy for the assessment of diastolic inflow [62]. CMR has the potential to evaluate the diastolic function during both active and passive stages with the additive value of assessment of myocardial velocities [63], providing insights unavailable by other non-invasive imaging techniques. Recently, a novel CMR-derived index -diastolic volume recovery, calculated as the percentage proportion of diastole required for recovery of 80% stroke volume, has been shown to give the best result, compared with echocardiography for the detection of diastolic dysfunction [64]. Additionally, atrial size is an independent factor associated with CV morbidity and mortality [65] and the possibility of atrial fibrillation [66]. Assessment of atrial volumes by echocardio-graphy is inaccurate, due to geometric assumptions about their shape [67]. In contrary, CMR offers an accurate and reproducible measurement of atrial volumes and recently reference values of left atrial volume by CMR became available [68]. However, until now, due to high availability and temporal resolution of echocardiography, CMR has not been used as a routine tool for diastolic function evaluation.

#### Differentiation of various causes of LVH

The differentiation of aetiology of LVH is intriguing, because various forms of LVH may present with overlapping phenotypes. Various myocardial abnormalities including infiltrative diseases, hypertrophic cardiomyopathy (HCM), Fabry’s disease, cardiac sarcoidosis, aortic stenosis and athlete’s heart can be presented with LVH.

CMR is superior to echocardiography for HCM diagnosis, by identifying areas of segmental hypertrophy (ie, anterolateral wall or apex) not reliably visualized by echocardiography (or underestimated in extent). High-risk HCM subgroups, identified with CMR, include those with thin-walled scarred LV apical aneurysms (which prior to CMR imaging remained usually undetected), end-stage systolic dysfunction and massive LV hypertrophy. CMR observations also suggest that the cardiomyopathic process in HCM is more diffuse than previously observed, extending beyond the LV myocardium to include thickening of the right ventricular wall as well as substantial morphologic diversity with regard to papillary muscles and mitral valve. These findings have implications for management in HCM undergoing invasive septal reduction therapy. Among HCM families, CMR has identified unique phenotypes of affected genetic status in the absence of LV hypertrophy, including myocardial crypts, elongated mitral valve leaflets and late gadolinium enhancement [69]. CMR may also raise suspicion of various infiltrative cardiomyopathies, such as cardiac amyloidosis, glyocogen/lysosomal storage diseases including Fabry’s, Danons, and AMP kinase diseases. The demonstration of nearly identically increased wall thickness in both the septum and LV free wall, by cine CMR, combined with diffuse subendocardial LGE is highly specific for cardiac amyloidosis. A similar pattern of concentric wall thickening with LGE confined to the basal inferolateral wall has been frequently reported in Fabry’s disease. CMR may suggest the aetiology of LVH; however, confirmatory diagnosis requires the identification of a disease-causing mutation by genetic testing or typical histopathology on cardiac biopsy [69].

CMR can be also helpful in detecting changes in serial measurements of LV wall thickness after treatment with antihypertensives, in which regression of hypertrophy supports the diagnosis of hypertensive cardiomyopathy. In hypertensive patients, CMR documented reduced ejection fraction/anteroseptal systolic strains and increased cardiac chamber volumes/LV wall stress, while in HCM, revealed supernormal ejection fraction, reduced LV wall stress/longitudinal systolic strain and fibrosis. Increased LV wall stress was the hallmark of hypertension, while HCM was characterized by reduced total longitudinal strain. Finally, athlete’s heart was distinguished from other types of hypertrophy by using the CMR-derived diastolic wall-to-volume ratio [70]. Although it needs further validation, a cut-off value of less than 0.15 mm × m2/ml has 99% specificity for sport-related LVH [70].

To conclude, although ECG has high specificity for the detection of LVH, it is unable to identify the type of hypertrophy. This is because the ECG thresholds are generally selected to optimize specificity. Finally, standard ECG criteria for LVH have low sensitivity for CMR LVH, and few false positives and more false negatives.Echocardiography, although widely available and cost effective, carries the significant disadvantages of operator and acoustic window dependency and restricted field of view. In contrary, the CMR capability to offer detailed and highly reproducible wall motion evaluation without assumptions and concurrent tissue characterisation can significantly facilitate the differential diagnosis of various diseases presented with LVH.

#### Fibrosis assessment

**Fig. 1:**
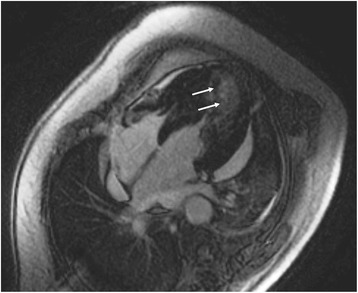
Four chamber LGE in a patient with extensive hypertrophy due to HCM. There are clear fibrotic areas in the interventricular septum, lateral wall of LV and apex, providing diagnostic and prognostic information about future cardiac events

**Fig. 2:**
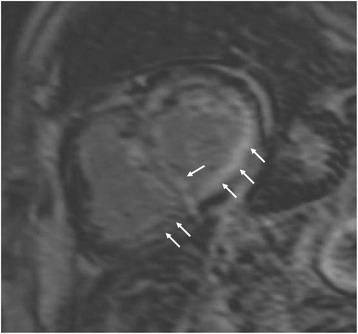
Short axis LGE in a patient with with amyloidosis. Evidence of amyloid depositions in both ventricles (*arrows*)

**Fig. 3:**
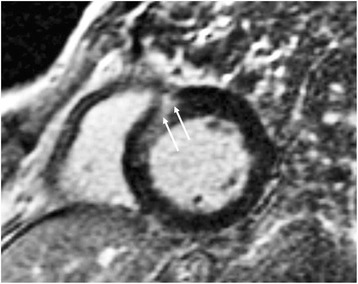
Short axis LGE in a patient with HHD and evidence of intranyocardial fibrosis in the interventricular septum

**Fig. 4:**
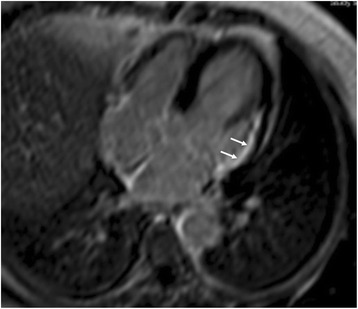
Subendocardial LGE in the lateral wall of LV, due to myocardial infarction in a patient with coronary artery disease

Myocardial fibrosis is the common end point of various pathologic processes in HHD and plays an important role in the development of diastolic dysfunction [71, 72]. The most robust approach to quantify replacement myocardial fibrosis is late gadolinium enhancement (LGE) [73]; it represents gadolinium enhancement in regions of fibrosis using T1-weighted images, taken 10–15 min after intravenous administration of gadolinium-based contrast agent. The pathophysiologic mechanism behind LGE includes a) expansion of extravascular volume in fibrotic myocardium, occupied by the extracellular distribution of contrast agent b) impaired kinetics of gadolinium due to vascular changes in fibrotic myocardium. Patchy LGE has been documented in approximately 50% of patients with LVH due to arterial hypertension [74], and was clearly distinguishable from the subendocardial LGE (Fig. 4), due to myocardial infarction. LGE can be also found in hypertrophic cardiomyopathy, and its quantification can differentiate those patients in high risk for sudden cardiac death [75] (Figs. 1, 2, 3 and 4).

Recent studies proved that the severity of diastolic dysfunction increases in parallel with the extent of fibrosis, assessed by LGE [76]. However, discrete LGE areas may be absent in HHD. Unfortunately, diffuse myocardial fibrosis can not be detected by LGE, because LGE relies on the signal intensity differences between fibrotic and normal myocardium and this is a serious drawback of the technique. This phenomenon has motivated the development of the technique of T1 mapping by which quantification of diffuse myocardial fibrosis can be achieved; T1 mapping represents an independent discriminator between HCM and hypertension, over and above extracellular volume fraction (ECV), LV wall thickness and indexed LVM [77]. There are data demonstrating significant differences in myocardial contrast accumulation between controls and HF patients using post-contrast T1 mapping with concurrent histologic data supporting that these changes reflect diffuse fibrosis. T1 mapping has the potential to be the end point in future trials, assessing antifibrotic treatment in HHD, without using serial endomyocardial tissue biopsies [4, 78, 79].

Finally, in well-controlled hypertensive patients, conventional CMR discovered significant underlying diseases (infarction, HCM), undetected by echocardiography. In these patients, T1 mapping revealed increased diffuse myocardial fibrosis, but this increase was small and only occurred with LVH [80]. However, in another study T1 mapping was an independent discriminator between HCM and hypertension, over and above extracellular volume fraction (ECV), LV wall thickness and LVM [81].

Although tissue biopsy is the gold standard for diagnosis of myocardial fibrosis, some circulating biomarkers have been also proposed for the non-invasive assessment of fibrosis. It was suggested that galectin-3 (Gal-3) is associated with myocardial histological and molecular parameters related to fibrosis and with the circulating biomarkers of the extracellular generation of mature fibril-forming collagen types I (C-terminal propeptide of procollagen type I, PICP) and III (N-terminal propeptide of procollagen type III, PIIINP) in two independent studies of hypertensive patients with heart failure (HF). However, data from various studies are rather contradictory. The excess of cardiac and systemic Gal-3 in HF patients of hypertensive origin was not associated with histological, molecular and/or biochemical parameters related to myocardial fibrosis in these patients [82]. Furthermore, a review of the literature about biomarkers showed that most of them lack proof for representing true myocardial fibrosis [83]. However, in another study, elevated serum levels of Gal-3 were in agreement with the degree of myocardial fibrosis assessed by LGE [84].

#### Ischemia detection

In HHD, coronary circulation changes take place, independently of occlusive atherosclerotic disease of epicardial coronary arteries. Furthermore, abnormalities in the coronary microcirculation, which accompany cardiac hypertrophy, play an important role in the pathophysiology of complications, attributed to LVH [85]. Although in clinical practice, the ECG is the first test to assess LVH [86, 87], false positives results are very common. Furthermore, a normal ECG can not exclude LVH [88]. Myocardial perfusion scintigraphy can potentially have a place in the detection of reduced coronary flow reserve in HHD. However, there are several technical issues with this technique in HHD, because any myocardial pathology, frequently assessed in these patients, can lead to abnormal images. Patients with HHD and concurrent LVH may have perfusion defects, unrelated to coronary artery disease (CAD). Their images can be false interpreted as evidence of myocardial ischemia, due to CAD [89, 90]. Furthermore, in HHD with LVH and/or microvascular disease, myocardial perfusion abnormalities were frequently documented in hypertensive patients with associated angiographically normal epicardial coronary arteries [90]. Stress echocardiography has a better specificity for detection of angiographically documented CAD [91]; however, the sensitivity of wall motion abnormalities for ischemia detection was significantly reduced, when LVH coexisted.

**Fig. 5:**
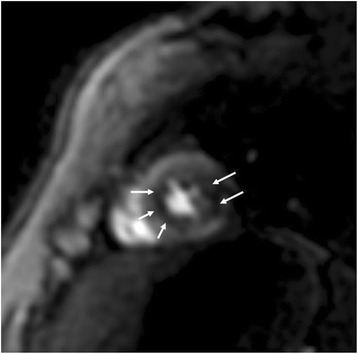
Diffuse subendocardial perfusion defect, detected by adenosine stress perfusion CMR, in a patient with HHD

Stress perfusion CMR permits the non-invasive differentiation between hemodynamically significant coronary artery stenosis and microvascular disease, due to hypertension and/or diabetes, based on high temporal and spatial resolution of the technique. In small vessel disease, the perfusion defects are usually diffuse, circumferential, affecting ≤1/3 of the wall thickness and have shorter persistence (≤ 5 heartbeats) compared with those in patients with significant epicardial coronary disease (Fig. 5) [92–95]. This differentiation is important for both treatment and prognosis [96, 97]. CMR can also identify coronary flow abnormalities in HHD with normal coronary arteries [98, 99]. Additionally, more than half of the scar detected by CMR-LGE was undetected by ECG [5, 6].

#### CMR indications in HHD

Clinical evaluation, ECG and echocardiography constitute the cornerstones for both diagnosis and follow up of HHD and remain the absolutely necessary examinations for HHD patients. However, a CMR evaluation is indicated if there is a) an inconclusive echocardiogram, b) a mismatch between clinical evaluation, ECG and echocardiography and c) severe, rapidly progressive LVH. Finally, due to high reproducibility, it is the ideal tool for clinical trials. In more details, the CMR indications in HHD are presented in Table 1.

**Table 1:** Potential CMR indications in hypertensive heart disease

Aggressive, rapidly progressive hypertension	
Poor acoustic window	
HHD and stroke (to exclude the potential of aortic plaques)	
Differential diagnosis of the etiology of LV hypertrophy	
Quantification of concurrent aortic valve regurgitation	
Evaluation of aortic tree anatomy and exclusion of potential renovascular disease	
Differential diagnosis of the etiology and pattern of fibrosis	
Documentation of microvascular cardiac disease	
Evaluation of treatment	
Reduction of the clinical studies cost (smaller patients’ sample for drug validation is needed by CMR)	

## Conclusions

HHD is the commonest cause of cardiovascular death with detrimental impact on patients’ morbidity and mortality and also health care costs. Although detailed guidelines determining the clinical indications of CMR in hypertension are still missing, CMR can provide early and highly reproducible evaluation of LVH and remodelling, not available by any other non-invasive technique; furthermore, the capability to perform tissue characterisation facilitates the early diagnosis and better risk stratification of micro-, macro-vascular ischemia and fibrosis, commonly found in hypertensive patients, with potentially high impact on their treatment and also on health care costs.

## Key points

1. Hypertensive heart disease (HHD) includes
  a) Left ventricular hypertrophy (LVH),
  b) congestive heart failure (CHF),
  c) ischemic heart disease (IHD)
2. ECG evaluation of LVH lacks sensitivity and specificity, particularly in young male patients
3. 2D Echocardiographic assessment of LV mass (LVM) is useful in patients with severe LVH; however, it has high interobserver/interstudy variability
4. LV mass assessed by RT-3DE correlates with CMR better than that determined by 2DE; however, underestimation and variability remain challenges in patients with ischemic cardiomyopathy
5. CMR offers:
  a) High reproducibility of measurements
  b) Easier and faster evaluation of treatment
  c) Evaluation of intramyocardial function
  d) Evaluation of Diastolic dysfunction
  e) Differentiation of various causes of LVH
  f) Fibrosis assessment
  g) Ischemia detection

## Abbreviations

2D E: 2 Dimentional Echocardiography
Am: Atrial contraction, diastolic mitral annular velocity
CAD: Coronary artery disease
CHF: Congestive heart failure
CMR: Cardiovascular Magnetic Resonance
Ea: Early diastolic mitral annular velocity
ECG: Electrocardiogram
EF: Ejection fraction
FI: M-mode fundamental imaging
Gal-3: Galectin 3
HCM: Hypertrophic Cardiomyopathy
HF: Heart failure
HFpEF: Heart failure with preserved ejection fraction
HHD: Hypertensive heart disease
HI: M-mode harmonic imaging
IHD: Ischemic heart disease
IHD: Ischemic heart disease
IVS: Intra-ventricular septal
LGE: Late gadolinium enhancement
LV: Left ventricle
LVH: Left ventricular hypertrophy
LVM: Left ventricular mass
NSF: Nephrogenic fibrosis syndrome
PICP: C-terminal propeptide of procollagen type I
PIIINP: N-terminal propeptide of procollagen type III
RT-3DE: 3 Dimentional Echocardiography
RV: Right ventricle
SSFP: Steady-state free precession
STIR: T2 T2-weighted short-tau inversion recovery
